# Replication of Putative Susceptibility Loci from Genome-Wide Association Studies Associated with Coronary Atherosclerosis in Chinese Han Population

**DOI:** 10.1371/journal.pone.0020833

**Published:** 2011-06-16

**Authors:** Fang Xie, Xun Chu, Hong Wu, Weiwei Sun, Min Shen, Lin Yang, Ying Wang, Yi Wang, Jinxiu Shi, Wei Huang

**Affiliations:** 1 Ruijin Hospital, School of Medicine, Shanghai Jiaotong University, Shanghai, China; 2 Shanghai-MOST Key Laboratory of Health and Disease Genomics, Department of Genetics, Chinese National Human Genome Center, Shanghai, China; 3 Department of Cardiology, Changhai Hospital, The Second Military Medical University, Shanghai, China; The University of Hong Kong, Hong Kong

## Abstract

**Background:**

Coronary atherosclerosis, the main cause of cardiovascular disease, is a progressive disease. Recent Genome Wide Association Studies (GWASs) discovered several novel loci associated with coronary artery disease (CAD) or its main complication myocardial infarction (MI). In this study, we investigated the associations between previously reported CAD- and MI-associated variants and coronary atherosclerosis in Chinese Han population.

**Methodology/Principal Findings:**

We performed a case-control association study with 2,335 coronary atherosclerosis patients and 1,078 controls undergoing coronary angiography of Chinese Han from China. Fourteen single nucleotide polymorphisms (SNPs), located at 1p13.3, 1q41, 2q36.3, 6q25.1, 9p21.3, 10q11.21 and 15q22.33, were genotyped in our sample collection. Six SNPs at 9p21 were associated with coronary atherosclerosis susceptibility (P_trend_<0.05) and rs10757274 showed the most significant association (P = 2.38×10^−08^, OR = 1.34). These associations remained significant after adjustment for multiple comparisons. Rs17465637 at 1q41 (P_trend_ = 6.83×10^−03^, OR = 0.86) also showed significant association with coronary atherosclerosis, but the association was not significant after multiple comparisons. Additionally, rs501120 (P = 8.36×10^−03^, OR = 0.80) at 10q11.21 was associated with coronary atherosclerosis in females, but did not show association in males and all participants. Variants at 1p13.3, 2q36.3, 6q25.1 and 15q22.33 showed no associations with coronary atherosclerosis and main cardiovascular risk factors in our data.

**Conclusions/Significance:**

Our findings indicated variants at 9p21 were significantly associated with coronary atherosclerosis in Han Chinese. Variants at 1q41 showed suggestive evidence of association and variants at 10q11.21 showed suggestive evidence of association in females, which warrant further study in a larger sample.

## Introduction

Coronary atherosclerosis is a progressive disease and the potential consequences in atherosclerosis include: coronary artery disease (CAD) and its main complication myocardial infarction (MI). CAD and MI are leading causes of death and disability worldwide and have a rapidly increasing incidence in developing countries [1]. Epidemiological studies have revealed that both genetic and environmental risk factors contributed to the pathogenesis of atherosclerosis [2]. However, the molecular mechanism of atherogenesis, including formation, proliferation and atheroma rupture, has not yet been clarified.

Biological complexity of atherosclerosis implies involvement of a large number of genes and their functional variants in its pathogenesis [3]. During the past five decades, large-scale epidemiological studies and genetic association analysis have identified multiple risk factors and susceptibility genes for coronary atherosclerosis [4]. Variants of several functionally important genes, including *ApoE* [5] and *LDLR* [6], have been implicated in susceptibility to coronary atherosclerosis in general population. With improved genotyping technologies and the completion of the human HapMap project, Genome-Wide Association Studies (GWASs) have recently become an important research method in genetics study. Recent GWASs and meta-analysis in CAD and MI identified several new susceptibility loci [7], [8], [9], [10], [11], [12]. Among these loci, the strongest association signals were on chromosome 9p21.3, which were also correlated with stroke, abdominal aortic and intracranial aneurysms in several other cohorts [13]. Variants on chromosome 1p13.3 was also found to be significantly associated with CAD and LDL cholesterol concentration in recent GWASs [14], [15], [16], [17], [18], reinforcing the mechanistic relationship between the variability in LDL levels and CAD risk [19].

Most of these GWASs were conducted in Caucasian populations, and several replication studies have been performed in Chinese population from China. Rs2383206 and rs2383207 were investigated association with CAD in 1,360 cases and 1,360 gender-matched controls of Chinese Han, and only rs2383207 locus was found to be significantly associated with CAD [20]. The associations of rs10757274, rs2383206, and rs10757278 were investigated with MI in Chinese Han subjects by conducting a hospital-based case–control study (432 cases and 430 controls), and all the three SNPs showed association with MI [21]. Rs1333049 were found to be an independent determinant for coronary plaque progression in 1,034 non-diabetic patients but not in 1,012 type 2 diabetic mellitus (T2DM) patients from Chinese Han [22]. Rs2383206, rs1004638 and rs10757278, in a strong linkage disequilibrium (LD) block, were investigated in 510 CAD patients, 558 patients with ischemic stroke and 557 shared controls of Chinese Han, and showed significant associations with CAD and weak association with Ischemic Stroke [23]. Four SNPs at 9p21 were genotyped in 425 MI patients, 687 patients with ischemic stroke, and 1,377 healthy controls recruited from Chinese population residing in Taiwan, and the result showed Genetic variations in the 9p21 region are associated with MI but not with stroke [24]. All the above-mentioned studies in Chinese population were limited to 9p21 variants.

Here, we undertook a replication study in a large cohort of coronary atherosclerosis patients and controls from Chinese Han population. We examined 14 SNPs located in seven chromosome regions which showed strong or moderate associations with CAD and MI from recent GWASs [7], [8], [9], [10]. Among these new risk loci, variants at 9p21.3 have been firmly validated in the follow-up replication studies from different populations [20], [25], [26], [27]. Since the initial GWASs were conducted in Caucasian populations of which the LD pattern is quite different from that of Chinese, and the most significant associated SNP was also different in separate studies, fine-mapping of 9p21.3 in Chinese population is needed. We selected eight SNPs, which represent the most associated and independent SNPs at 9p21.3 in the previous studies, to perform a limited investigation of the associations of the region in Han Chinese.

## Results

Clinical and biochemical characteristics according to the presence of significant coronary stenoses are summarized in Table 1. As anticipated, the prevalence of established cardiovascular risk factors was higher in coronary atherosclerosis cases than that in controls. Overall, the characteristics of our patients were typical for patients undergoing coronary angiography for the evaluation of coronary atherosclerosis, with a male preponderance (61.8%) and a high prevalence of T2DM (15.8%), hypertension (59.5%), smoking (30.1%) as well as drinking (10.7%). Notably, the levels of serum LDL cholesterol were significantly lower but HDL cholesterol levels were higher in patients with significant coronary stenoses than in patients without such lesions.

**Table 1:** Clinical and biochemical characteristics of participants included in the current study.

Variable	Controls (n = 1078)	Cases (n = 2335)	P value
Men (%)	50.8	66.9	<0.001
Ever and current smokers (%)	22.9	33.4	<0.001
Ever and current drinkers (%)	9.3	11.4	0.069
Hypertension(%)	51.1	63.7	<0.001
Diabetes (%)	7.0	19.9	<0.001
Age(years): mean ± SD	60.4±10.3	65.4±10.2	<0.001
Height (CM): mean ± SD	164.98±7.90	166.37±7.61	<0.001
Weight (KG): mean ± SD	67.00±11.23	67.96±10.60	0.015
BMI: mean ± SD	24.57±3.41	24.48±3.06	0.487
Total cholesterol (mmol/L): mean ± SD	4.66±1.00	4.77±1.22	0.009
Triglyceride (mmol/L): mean ± SD	1.64±0.92	1.76±1.10	0.001
HDL-C (mmol/L): mean ± SD	1.26±0.37	1.17±0.40	<0.001
LDL-C(mmol/L): mean ± SD	2.76±0.80	2.90±0.97	<0.001

SD, standard deviation: HDL-C, high-density lipoprotein cholesterol; LDL-C, low-density lipoprotein cholesterol.

**Figure 1:**
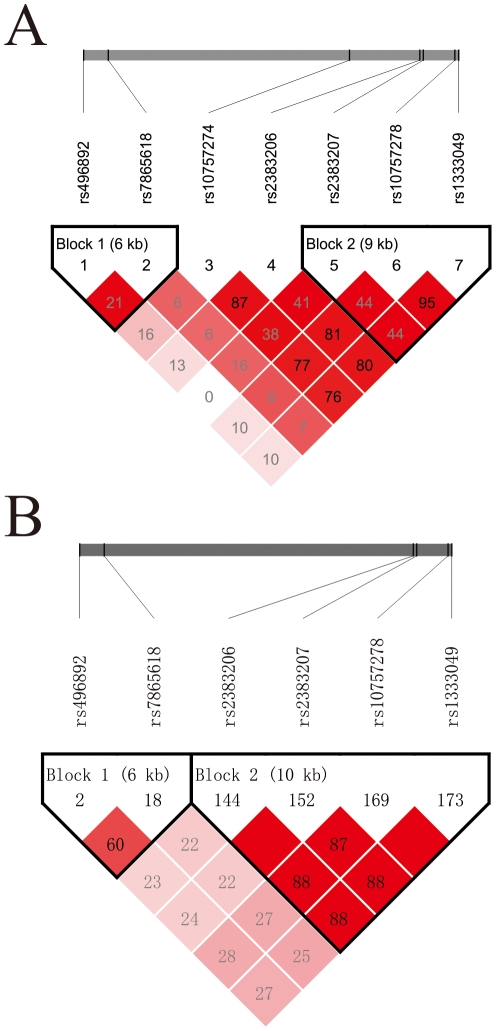
LD plot of SNPs in chromosome 9p21.3. (A) LD plot of seven SNPs in chromosome 9p21.3 of Chinese Han population from current study. The r^2^ values were estimated from data of 2,335 atherosclerosis patients and 1,078 controls enrolled in the current study; (B) LD structure of six risk SNPs at 9p21.3 in CEU population (Europeans) from the data of HapMap phaseII release 23. We constructed the plot with Haploview 4.2 [47], and r^2^ (×100) values were depicted in the diamonds. Blocks were determined using the default method of Gabriel *et al* [48].

Among the 14 SNPs, rs17228212 with a minor allele frequency (MAF) of less than 1% was removed from further association analysis. In addition, rs7044859 with Hardy-Weinberg equilibrium (HWE) of p<0.01 in controls were also eliminated from further analysis (Table 2). Twelve SNPs conformed to HWE were investigated association in 2,335 coronary atherosclerosis patients and 1,078 control subjects. The LD structure among seven SNPs conformed to HWE at 9p21.3 was examined by program Haploview 4.2 (Figure 1A).

**Table 2:** Genotyping data for 14 SNPs in 2,335 coronary atherosclerosis patients and 1,078 controls.

Chr	Position	SNP	Gene annotation	Allele (1/2)	Genotype of case			Genotype of control			MAF	P-HWE
					1/1	1/2	2/2	1/1	1/2	2/2		
1	109623689	rs599839	3′downstream of PSRC1	G/A	4	275	2015	5	141	885	0.073	1.00
1	220890152	rs17465637	Intron of MIA3	A/C	315	1014	930	171	485	380	0.400	0.44
2	226776324	rs2943634	Intergenic	A/C	15	360	1909	11	165	871	0.089	0.34
6	151294678	rs6922269	Intron of MTHFD1L	A/G	0	78	2232	0	39	1002	0.019	1.00
9	22008781	rs7044859	Intron of ANRIL, 5′upstream of CDKN2A/2B	T/A	229	440	1592	142	192	709	0.229	<0.01
9	22014351	rs496892	Intron of ANRIL, 5′upstream of CDKN2A/2B	A/G	270	1013	996	158	490	418	0.378	0.47
9	22021005	rs7865618	Intron of ANRIL, 5′upstream of CDKN2A/2B	G/A	22	428	1840	12	224	807	0.119	0.55
9	22086055	rs10757274	Intron of ANRIL, 5′upstream of CDKN2A/2B	G/A	569	1196	527	197	526	330	0.436	0.66
9	22105026	rs2383206	Intron of ANRIL, 5′upstream of CDKN2A/2B	G/A	583	1110	500	217	501	320	0.450	0.42
9	22105959	rs2383207	Intron of ANRIL, 5′upstream of CDKN2A/2B	A/G	197	931	1170	113	484	466	0.335	0.49
9	22114477	rs10757278	3′downstream of ANRIL, 5′upstream of CDKN2A/2B	A/G	507	1139	664	300	518	233	0.532	0.76
9	22115503	rs1333049	3′downstream of ANRIL, 5′upstream of CDKN2A/2B	G/C	506	1140	659	295	525	241	0.526	0.81
10	44073873	rs501120	5′upstream of CXCL12	G/A	282	1068	969	146	477	421	0.368	0.55
15	65245693	rs17228212	Intron of SMAD3	G/A	0	5	2301	0	1	1050	<0.001	1.00

1/2, minor allele/major allele; MAF, minor allele frequency; HWE, Hardy-Weinberg Equilibrium.

Association analysis of the 12 SNPs was performed by using genotype and Cochran-Armitage trend tests. Of the 12 SNPs, seven SNPs showed associations with coronary atherosclerosis: rs17465637 at 1q41 (P_trend_ = 6.83×10^−03^, OR = 0.86, 95% CI = 0.77−0.96), and six SNPs at 9p21: rs496892 (P_trend_ = 3.13×10^−03^, OR = 0.85, 95% CI = 0.76− 0.95), rs10757274 (P_trend_ = 2.38×10^−08^, OR = 1.3, 95% CI = 1.2−1.4), rs2383206 (P_trend_ = 2.77×10^−07^, OR = 1.3, 95%CI = 1.2−1.5), rs2383207 (P_trend_ = 1.54×10^−04^, OR = 0.81, 95% CI = 0.72−0.90), rs10757278 (P_trend_ = 6.52×10^−07^, OR = 0.77, 95% CI = 0.69−0.85), rs1333049 (P_trend_ = 8.57×10^−06^, OR = 0.79, 95% CI = 0.71−0.88) (Table 3). After accounting for the most significant SNPs rs10757274, no other SNPs at 9p21.3 were independently associated with coronary atherosclerosis in logistic regression analysis, suggesting rs10757274 could solely account for the association signal of this region (Table 4). All the seven SNPs remained significant after adjustment for age and sex. Rs599839 at 1p13.3 was not associated with coronary atherosclerosis in univariate analyses; however, it showed a nominal association after adjusting for age and sex (Table 3). We also investigated associations in a multivariate model including age, sex, rs17465637 at 1q41, rs599839 at 1p13.3 and rs10757274 at 9p21.3, and all three SNPs remained significant (P = 0.009 for rs17465637, P = 0.02 for rs599839, and P = 3.83×10^−07^for rs10757274).

**Table 3:** Association analyses of the selected SNPs in 2,335 coronary atherosclerosis patients and 1,078 controls.

Chr	Position	SNP	P _genotype_ *	P _trend_ *	OR (95% CI)	P _adjusted_^a^ *	OR (95% CI)^a^ *
1	109623689	rs599839	0.09	0.07	0.83 (0.68–1.02)	**0.02**	0.77 (0.62–0.96)
1	220890152	rs17465637	**0.02**	**6.83×10** ^−**03**^	0.86 (0.77–0.96)	**3.71×10** ^−**03**^	0.85 (0.76–0.95)
2	226776324	rs2943634	0.5	0.60	0.95 (0.79–1.14)	0.63	0.95 (0.79–1.16)
6	151294678	rs6922269	0.61	0.59	0.90 (0.61–1.33)	0.64	0.91 (0.60–1.37)
9	22014351	rs496892	0.01	**3.13×10^−03^**	0.85 (0.76–0.95)	**0.01**	0.87 (0.78–0.97)
9	22021005	rs7865618	0.13	0.05	0.85 (0.72–1.00)	0.14	0.88 (0.74–1.05)
9	22086055	rs10757274	**1.83×10** ^−**07**^	**2.38×10** ^−**08**^	1.34 (1.21–1.48)	**5.14×10** ^−**08**^	1.37 (1.22–1.53)
9	22105026	rs2383206	**1.21×10** ^−**06**^	**2.77×10** ^−**07**^	1.32 (1.19–1.46)	**4.36×10** ^−**07**^	1.33 (1.19–1.49)
9	22105959	rs2383207	**4.56×10** ^−**04**^	**1.54×10** ^−**04**^	0.81 (0.72–0.90)	**4.23×10^−04^**	0.81 (0.72–0.91)
9	22114477	rs10757278	**5.20×10^−06^**	**6.52×10^−07^**	0.77 (0.69–0.85)	**2.08×10^−06^**	0.77 (0.69–0.86)
9	22115503	rs1333049	**6.95×10^−05^**	**8.57×10^−06^**	0.79 (0.71–0.88)	**1.28×10^−05^**	0.79 (0.70–0.88)
10	44073873	rs501120	0.33	0.19	0.93 (0.84–1.04)	0.12	0.91 (0.81–1.02)

OR, odds ratio; 95% CI, 95% confidence interval.

a The adjusted P-value and OR in multiple logistic regression after adjustment for age and sex.

*P-value of <0.05 was considered statistically significant, and P <0.0036 was considered significant after Bonferroni correction for 14 SNPs.

**Table 4:** Logistic regression analysis of 9p21.3 associated with atherosclerosis in Chinese Han population.

Chr	rs	Position	OR (95% CI)	Single-locus test P	r^2^ with rs10757274	Single-locus test P when SNP added to rs10757274	Single-locus test P when rs10757274 added to SNP
9	rs496892	22014351	0.85 (0.76–0.95)	3.13×10^−03^	0.17	0.52	6.90×10^−07^
9	rs7865618	22021005	0.85 (0.72–1.00)	5.17×10^−02^	0.07	0.67	5.32×10^−07^
9	rs10757274	22086055	1.34 (1.21–1.48)	2.38×10^−08^	/	/	/
9	rs2383206	22105026	1.32 (1.19 - 1.46)	2.77×10^-07^	0.87	0.45	5.83×10^-03^
9	rs2383207	22105959	0.81 (0.72 - 0.90)	1.54×10^-04^	0.38	0.61	4.44×10^-05^
9	rs10757278	22114477	0.77 (0.69 - 0.85)	6.52×10^-07^	0.77	0.89	0.01
9	rs1333049	22115503	0.79 (0.71–0.88)	8.57×10^−06^	0.75	0.43	7.57×10^−04^

The single–locus tests were restricted to complete genotypes for each SNP in the separate region.

OR, odds ratio for the minor allele; 95% CI, 95% confidence interval; r^2^, the coefficient of LD between rs10757274 and the additional SNPs.

Previous studies have suggested sex-specific heritability of coronary atherosclerosis disease [28], [29]. To address this issue, we performed stratified association analysis by sex. Rs17465637 at 1q41 and rs496892 at 9p21, which showed association in all the participants, were associated with coronary atherosclerosis in the female subgroup but not in males. Rs501120 at 10q11.21 was associated with disease in female group (P_female_ = 7.53×10^−03^), but did not show association in males and all participants. Additionally, rs2383207 was associated with disease in the male subgroup but not in the females. We also performed association analysis between the selected SNPs and the known risk factors for coronary atherosclerosis (hypertension and diabetes). And we found seven SNPs at 9p21 were associated with susceptibility of atherosclerosis complicated by hypertension, and five SNPs in block 2 at 9p21 showed associations in non-hypertension subgroup (Figure 1A). In the cohort without diabetes, the distributions of rs599839 at 1p13.3, rs17465637 at 1q41 and seven SNPs at 9p21 showed significant differences between cases and controls. We found no evidence for association with the 6q25.1, 2q36.3 and 15q22.33 loci. The results were summarized in Table 5.

**Table 5:** Association analyses of the selected SNP and atherosclerosis with risk factors.

Chr	Position	SNP	P _female_	P _male_	P _hypertension_	P _non-hypertension_	P _diabetes_	P _non- diabetes_
1	109623689	rs599839	0.30	0.06	0.71	0.05	1.00	**0.04**
1	220890152	rs17465637	**0.02**	0.10	0.05	0.07	0.11	**0.01**
2	226776324	rs2943634	1.00	0.57	0.61	1.00	0.24	0.84
6	151294678	rs6922269	0.55	0.18	0.58	1.00	1.00	0.68
9	22014351	rs496892	**0.03**	0.07	**1.38×10^−04^**	0.84	0.85	**2.85×10^−03^**
9	22021005	rs7865618	0.82	0.06	**3.38×10^−03^**	0.70	0.39	**0.03**
9	22086055	rs10757274	**2.18×10^−03^**	**1.45×10^−05^**	**4.52×10^−04^**	**4.03×10^−05^**	0.16	**2.54×10^−07^**
9	22105026	rs2383206	**6.04×10^−03^**	**1.86×10^−05^**	**7.64×10^−04^**	**5.69×10^−05^**	0.13	**1.48×10^−06^**
9	22105959	rs2383207	0.05	**2.55×10^−03^**	**0.03**	**1.78×10^−03^**	1.00	**2.81×10^−05^**
9	22114477	rs10757278	**0.01**	**3.79×10^−05^**	**6.53×10^−04^**	**3.17×10^−04^**	0.24	**8.66×10^−07^**
9	22115503	rs1333049	**0.02**	**1.95×10^−04^**	**3.08×10^−03^**	**9.00×10^−04^**	0.33	**1.55×10^−05^**
10	44073873	rs501120	**8.36×10^−03^**	0.67	0.77	0.09	0.52	0.23

Finally, we examined serum lipid levels with respect to genotypes of the twelve variants. In univariate analyses, rs17465637 at 1q41 was associated with HDL cholesterol (P = 0.018); no other SNPs showed association with serum LDL cholesterol and HDL cholesterol. SNP rs599839 at 1p13.3 was associated with plasma levels of total and LDL cholesterol in previous studies [14], [15], [16], [17]; however, showed no evidence of association in our data (Table 6).

**Table 6:** Distribution of serum lipid levels in the study according to genotypes of rs599839 and rs17465637.

	Total cholesterol (mmol/l) ± SD	Triglycerides (mmol/l) ± SD	HDL-C (mmol/l) ± SD	LDL-C (mmol/l) ± SD
rs599839				
GG (9)	4.96±0.77	1.73±0.68	1.29±0.26	2.94±1.03
GA (416)	4.66±1.16	1.64±1.07	1.21±0.33	2.83±0.97
AA (2900)	4.73±1.16	1.73±1.04	1.20±0.40	2.85±0.91
P value	0.39	0.27	0.75	0.85
rs17465637				
AA (486)	4.78±1.10	1.68±0.99	1.25±0.59	2.84±0.96
AC (1499)	4.69±1.19	1.70±1.03	1.19±0.33	2.83±0.90
CC (1310)	4.78±1.13	1.78±1.09	1.19±0.35	2.88±0.92
P value	0.12	0.09	**0.02**	0.35

SD, standard deviation; HDL-C, high-density lipoprotein cholesterol; LDL-C, low-density lipoprotein cholesterol.

## Discussion

Coronary atherosclerosis, the primary cause of CAD, is a progressive disease. MI is the last step in the development of CAD, and thrombogenic factors ultimately determine whether or not infarction occurs [11], [12]. In the past few years, a number of novel susceptibility genes of CAD or MI were identified using GWASs. In particular, several SNPs identified by the WTCCC, McPherson et al and Helgadottir et al met the criteria for genome-wide association [7], [8], [10], [30]. Other studies found associations of these variants with myocardial infarction [25], [27], [31]. Potential associations between variants on these risk loci and angiographically characterized coronary atherosclerosis are unknown, although some investigations had been made [32]. We therefore aimed at investigating the association of these reported variants with coronary atherosclerosis in a large Chinese population consisted of well-characterized patients and controls. Our data validated the variation at 9p21 for association with coronary atherosclerosis, and provided evidence for the association of rs17465637 at 1q41.

Variants at 9p21 showed association with genome-wide significance in our Chinese Han collection. Rs10757274 was the most significant SNP among the seven SNPs in this region, and after accounting for rs10757274, no other SNPs at 9p21.3 showed association signal. Rs10757274 was also the most significant SNP in the GWAS by McPherson et al. [8]. However, rs1333049 was most significantly associated with CAD risk in the GWASs by the WTCCC and the German Cardiogenics Consortium [9], [10], and rs10757278 was the lead SNP in the GWAS of MI by Helgadottir et al. [7]. Indeed, rs1333049 and rs10757278 were in complete LD in both CEU and CHB data from HapMap, but were in low LD (r^2^ = 0.32) in the YRI data from HapMap. The LD between rs1333049 and rs10757274 were also high in both CEU and CHB data from HapMap (r^2^>0.89), as well as in our study (r^2^ = 0.76) and previous ones (r^2^ = 0.89) [33] (Figure 1A–B). Since the LD in this critical region is strong, using a population with low LD may help in localizing the association signal to the causal variants and therefore aid downstream analysis in future study.

The nearest described protein coding genes of these risk variants within chromosome 9p21 are *CDKN2A/2B* which encode inhibitors of *CDK4/CDK6*. The CDKN2A gene generates two transcripts derived from the alternative first exons, E1α and E1β, which incorporate exon 2 and 3 encoding p16/CDKN2A and p14/ARF, respectively [34]. A fourth gene, MTAP, is located further upstream in close proximity. All these genes play roles in cancer, cell-cycle control, apotosis and aging; however, their roles in the pathogenesis of atherogenesis are not clear. A new discovered large antisense noncoding RNA (designated antisense noncoding RNA in the INK4 locus, *ANRIL*) embedded in these genes, with a first exon located in the promoter of the p14/ARF gene and overlapping the two exons of *p15/CDKN2B* [35]. Previous studies showed that *ANRIL* was expressed in atherosclerotic tissue [36] and the expression of *ANRIL* was coordinated with that of *p14/ARF* and possibly with *p16/CDKN2A* and *p15/CDKN2B* in both physiological and pathological conditions [35], [37], suggesting that it might regulate the expression of these genes. The expression of *ANRIL* transcripts (EU741058 and NR_003529) in atherosclerotic plaque tissue was directly correlated with severity of atherosclerosis; however, no such correlation was found in *CDKN2A*, *CDKN2B* and *MTAP* [32]. Taken together, *ANRIL* might play a role in the pathogenesis of atherosclerosis. On the other hand, targeted deletion of the 9p21 non-coding interval in mice provided direct evidence that the risk interval has a pivotal role in regulation of cardiac *CDKN2A/B* expression, and suggested that this region affects CAD progression by altering the dynamics of vascular cell proliferation [38].

On chromosome 1q41, SNP rs17465637 was associated with coronary atherosclerosis in our study (P_trend_ = 6.83×10^−03^, OR = 0.86, 95% CI = 0.77−0.96) and also showed moderate association with HDL cholesterol (P = 0.02). Rs17465637 was firstly reported to be associated with CAD in a GWAS by samani et al. [9]. And then, it was reported to be associated with early-onset MI in the GWAS by Myocardial Infarction Genetics Consortium [39] and the association with MI was replicated in a Japanese sample [31]. However, in a large-scale association study from nine European studies, rs17465637 showed no significant association with CAD; instead, a SNP (rs3008621) in an adjacent haplotype block showed significant association [19]. Both rs17465637 and rs3008621 were located in the intron region of the melanoma inhibitory activity family, member 3 (*MIA3*) gene (also known as *ARNT* or *TANGO*). The MIA3 gene, coding for a 14 kDa protein of so far unknown function, was originally identified as a new member of MIA gene family. *MIA3* is widely expressed in vivo in contrast to the highly restricted expression pattern for the other family members [40]. Arndt et al. observed that *MIA3* expression was induced after adhesion of human monocytic cells to the substrate and found that recombinant MIA3 protein could reduce attachment of human monocytic cells to fibrinogen, ICAM-1 and human microvascular endothelial cells (HMECs). Additionally, the migrating capacity of premonocytic cells through fibrinogen or HMECs was increased after stimulation of these cells with recombinant *MIA3*. These results suggested that *MIA3* reduced the attachment to fibrinogen or other cell adhesion molecules [41]. This process is fundamental for the formation and progression of atherosclerotic plaque and also for plaque instability, which might play an important role in the development of coronary atherosclerosis.

Rs501120 at 10q11.21 lies upstream of the CXCL12 gene which codes for stromal cell-derived factor-1 (SDF-1), a member of the family of chemoattractant cytokines known as chemokines and is the ligand for cell-surface chemokine receptor 4. *SDF-1* has high expression in smooth muscle cells, endothelial cells, and macrophages in human atherosclerotic plaques, and was reported to be involved in the induction of platelet aggregation [42]. The combined analysis of The WTCCC study and the German MI study revealed that rs501120 at 10q11.21 was associated with CAD for the first time [9]. In 11,550 CAD cases and 11,205 controls from 9 European studies, rs501120 was replicated association with CAD and showed a stronger association in women than in men [19]. In our study, the SNP was associated with coronary atherosclerosis in women but not in man and all participants (P_all_ = 0.19, P_female_ = 8.36×10^−03^, P_male_ = 0.67). These results suggested that rs501120 might affect the pathology of atherosclerosis with sex difference, and the mechanism requires further investigation.

SNP rs599839, on chromosome 1p13.3, has recently been reported to be associated with CAD [9], [19] and LDL-cholesterol in several GWASs [14], [15], [16], [17], and the association with lipid concentrations were replicated in a Japanese population [43]. In univariate analyses of our study, SNP rs599839 was not associated with coronary atherosclerosis as well as serum lipids level; however, the association appeared significant after adjusting for age, sex and other significant SNPs in multivariate analyses. Assuming the prevalence of 0.50 and using a significance level of 0.05, our study had only 24.6% power to detect association with rs599839 (MAF of 7.3%) in 2,335 CAD patients vs. 1,078 controls. An even larger-scale case-control study in Chinese Han population should be performed to evaluate the association of rs599839 to CAD susceptibility and lipid serum lipids level.

Variants at 2q36.3, 6q25.1, 10q11.21 and 15q22.33 showed no associations in our data, and the following reasons might explain for the lack of association with atherosclerosis. First, the minor allele frequency of certain SNPs (rs6922269 and rs17228212) were low (MAF <5%) in Chinese population, and the sample size of our study was moderate, which has limited our power to detect the association. Therefore, further study with even larger sample size was needed to assess the associations. Second, previous GWASs might uncover risk alleles in these regions important for populations of Caucasians descent which may not contribute much to risk in Chinese due to different environment or other gene interactions, and therefore we did not replicated the associations of these region using the SNPs uncovered previously.

In conclusion, we have confirmed genetic associations for coronary atherosclerosis with 9p21. Additionally, our data showed suggestive evidence of association at 1q41 and suggestive evidence of association at 10q11.21 in females, which warrant further study in a larger sample. These findings provided a strong foundation for further investigation of these loci as risk factors for coronary atherosclerosis in Chinese Han population.

## Materials and Methods

### Ethics Statement

Approval to undertake this study was granted by the Ethics Review Committee of the Chinese National Human Genome Center at Shanghai and was conducted according to the Declaration of Helsinki Principles. Written informed consent was obtained from each recruited subjects.

### Study Populations

A total of 3,413 individuals were included in this study, consisting of 2,335 atherosclerosis patients and 1,078 unrelated controls free of atherosclerosis. Samples were collected from the cardiovascular care units of three hospitals in Shanghai. To reduce the potential confounding from ethnic backgrounds, we only enrolled people with self-reported origin of central Han Chinese, including indigenous people from Shanghai, Zhejiang Province, Jiangsu Province and Anhui Province. Recent analyses by Genome-wide SNP variation have shown that the central Han Chinese could be regarded as one single homogenous population [44], [45]. The diagnosis of coronary atherosclerosis was made on the basis of coronary angiography. Consensus diagnosis of coronary atherosclerosis was performed by two experienced doctors, who carefully evaluated the status by medical history, stenosis status and physical examination. Individuals with at least 50% atherosclerosis occlusion in more than two branches of the coronary artery were included as cases in the current study. All controls were individuals without coronary sterosis. Those with coronary myocardial bridge were excluded from the study. At enrollment, anthropometric measures, medication usage and family history data were collected from each subject by a trained interviewer. The demographic and risk factor information of all case and control samples were summarized in Table 1. Genomic DNA of all samples was isolated from whole blood using FlexiGene DNA Kit (Qiagen, Valencia, CA, USA).

### SNP selection and genotyping

Fourteen SNPs associated with CAD or MI from recent GWASs were studied, including those identified by McPherson et al (rs10757274 and rs2383206 at 9p21.3) [8], Helgadottir et al (rs2383207 and rs10757278 at 9p21.3) [7], Samani et al (rs599839 at 1p13.3, rs17465637 at 1q41, rs2943634 at 2q36.3, rs6922269 at 6q25.1, rs501120 at 10q11.21, rs17228212 at 15q22.33 and four tag SNPs of two blocks at 9p21.3 including: rs7044859, rs496892, rs7865618 and rs1333049) [9].

Eight SNPs (rs599839, rs6922269, rs10757278, rs2383206, rs7865618, rs501120, rs10757274 and rs17228212) were genotyped using SNPStream (Beckman Coulter, Fullerton, CA). Primer design for PCR and single base extension (SBE) was performed with Beckman Coulter Autoprimer software. Another six SNPs (rs17465637, rs2943634, rs1333049, rs496892, rs2383207 and rs7044859) were genotyped using TaqMan chemistry (Applied Biosystems). TaqMan genotyping assays with probes labeled with the fluorophores FAM and VIC were purchased from Applied Biosystems. The Universal PCR Master Mix from Applied Biosystems was used in a 5 µl total reaction volume with 10 ng DNA per reaction. Allelic discrimination was measured automatically on ABI Prism 7900HT (Applied Biosystems) with Sequence Detection Systems 2.1 software (auto caller confidence level, 95%). To evaluate the concordance of the two platforms, we selected rs1333049 to be re-genotyped in 100 randomly selected samples by using the SNPStream system. The concordance rate between the genotypes from the TaqMan and the SNPStream was 99%.

### Statistical analysis

Genotype distributions were evaluated for departure from HWE by Plink software [46] (version 1.07, http://pngu.mgh.harvard.edu/purcell/plink/). We performed genotype and Cochran-Armitage trend tests to assess genotype-phenotype association using Plink software. Allele frequencies for cases and controls were used to calculate the Odds Ratio (OR) and the 95% Confidence Interval (CI). Conditional logistic regression was performed to assess whether the most significant SNP in the associated region was sufficient to model the association. Multiple logistic regression was used to evaluate if each SNP was independently associated with CAD and serum lipid levels when adjusted for age and sex. Continuous data were expressed as mean ± standard deviations (SD) and independent-samples t-test was employed to analyze differences between two study groups. A two tailed P-value of <0.05 was considered statistically significant, whereas a value of corrected P<0.0036 was considered significant after Bonferroni correction for 14 SNPs. The software used for statistical calculations was the SPSS 15.0 (SPSS Inc., Chicago, IL, USA) unless specified.
